# CoronaVac (Sinovac) COVID-19 vaccine-induced molecular changes in healthy human serum by infrared spectroscopy coupled with chemometrics

**DOI:** 10.3906/biy-2105-65

**Published:** 2021-08-30

**Authors:** Ayca DOGAN, Rafig GURBANOV, Mete SEVERCAN, Feride SEVERCAN

**Affiliations:** 1 Department of Physiology, Faculty of Medicine, Altinbaş University, İstanbul Turkey; 2 Biotechnology Application and Research Center, Bilecik Şeyh Edebali University, Bilecik Turkey; 3 Department of Electrical and Electronics Engineering, Faculty of Engineering and Natural Sciences, Altınbaş University, İstanbul Turkey; 4 Department of Biophysics, Faculty of Medicine, Altınbaş University, İstanbul Turkey

**Keywords:** CoronaVac-Sinovac, vaccine, COVID-19, Attenuated total reflection-Fourier Transform Infrared (ATR-FTIR), Fourier Transform Infrared (FTIR) spectroscopy, multivariate analysis

## Abstract

From the beginning of the COVID-19 coronavirus pandemic in December of 2019, the disease has infected millions of people worldwide and caused hundreds of thousands of deaths. Since then, several vaccines have been developed. One of those vaccines is inactivated CoronaVac-Sinovac COVID-19 vaccine. In this proof of concept study, we first aimed to determine CoronaVac-induced biomolecular changes in healthy human serum using infrared spectroscopy. Our second aim was to see whether the vaccinated group can be separated or not from the non-vaccinated group by applying chemometric techniques to spectral data. The results revealed that the vaccine administration induced significant changes in some functional groups belonging to lipids, proteins and nucleic acids. In addition, the non-vaccinated and vaccinated groups were successfully separated from each other by principal component analysis (PCA) and linear discriminant analysis (LDA). This proof-of-concept study will encourage future studies on CoronaVac as well as other vaccines and will lead to make a comparison between different vaccines to establish a better understanding of the vaccination outcomes on serum biomolecules.

## 1. Introduction

The COVID-19 pandemic, also known as the coronavirus pandemic, which started in December 2019, is an ongoing global pandemic of coronavirus disease 2019 (COVID-19) caused by severe acute respiratory syndrome coronavirus 2 (SARS-CoV-2). The coronavirus pandemic has been continuously rising, spreading and infecting millions worldwide, causing hundreds of thousands of deaths. Since then, several vaccines have been developed and vaccination started in several countries with the approval of emergency use. One of those vaccines is CoronaVac (Sinovac) COVID-19 vaccine. Vaccination started in Turkey and some other countries at the beginning of January, 2020 with this inactivated vaccine. One of the advantages of this vaccine is that it can be transported and kept at 2–8 °C. 

Turkey was one of the countries whose phase III clinical trials were performed, and vaccination started with CoronaVac. Hacettepe University announced the final phase III results, first on 3 March 2021 and last stage trials at the beginning of July 2021. In total, trials were conducted with the participation of more than 10.000 people in Turkey and showed an efficacy of 83.5% against symptoms, and the vaccine prevented hospitalization and severe illness as 100% (Akova and Unal, 2021). 

While we have been working on healthy human serum samples for a human rare disease project by Fourier-transform infrared (FTIR) spectroscopy, we have noticed that the infrared spectra of healthy human serum samples show some differences between vaccinated and non-vaccinated ones. To find out these differences, which may be important to understand the effect of vaccination on biomolecules, we used attenuated total reflection Fourier-transform infrared (ATR-FTIR) spectroscopy. FTIR spectroscopy, especially ATR-FTIR spectroscopy coupled with multivariate analysis methods, is a very good candidate to determine molecular changes induced by external effects such as environment, chemicals and diseases. When ATR mode is used, a very small amount of sample, for example, one drop of biofluids, is directly placed on top of the crystal, and, with the improved infrared spectrometers, noise-free spectral data are collected in a very short time. In addition, FTIR spectroscopy is a rapid, cost-effective, easy-to-use, non-destructive, and operator-independent technique. It was also shown that this technique is independent of whether transmission or reflection mode is used (Gok et al., 2016). These properties make this technique a perfect candidate for translation from bench to clinic. Pathological situations induce structural and functional changes in molecules of biological systems. These changes cause variations in vibrational energy levels, which can be detected by infrared spectroscopy (Kraft et al., 2009; Severcan and Haris, 2012; Mordechai et al., 2017; Yonar et al., 2018; Rai et al., 2018). Up to now, complex and large infrared spectral data combined with multivariate analysis methods were successfully employed in diagnosis of several diseases such as different cancer types involving lung and mesothelioma (Abbas et al., 2018), breast (Backhaus et al., 2010), bladder (Gok et al., 2016), colon cancer (Kaznowska et al., 2017), diabetes (Toyran et al., 2006), neurological diseases such as multiple sclerosis (Yonar et al., 2018) and Alzheimer (Mordechai et al., 2017) and infectious diseases such as COVID-19 (Barauna, 2021; Zhang et al., 2021). Tissue sections, any cytological and histological samples or biofluids can be studied by infrared spectroscopy. In this context, biofluids such as serum, plasma, saliva and urine are ideal candidates for early detection of a wide range of diseases, since they are quite easily accessible and highly informative bioﬂuids (Barauna, 2021; Zhang et al., 2021). 

In the current “proof of concept study”, we aimed to determine the structural and contextual effects of the CoronaVac vaccine on healthy serum biomolecules such as lipids, proteins, and nucleic acids. To achieve this, we used ATR-FTIR spectroscopy coupled with unsupervised and supervised chemometric techniques. The information derived from this proof of concept study will give us some idea of whether this vaccine induces biological alterations at a molecular level or not. Accordingly, it will correctly direct researchers in ongoing and future human research studies and will contribute to the literature about the effectiveness of the CoronaVac vaccine.

## 2. Material and methods

### 2.1. Sample information and sample collection 

This study is carried out as a control group of a large scale project titled “Evaluation of Infrared Spectroscopy in the Molecular Characterization and Diagnosis of Myasthenia Gravis as a Novel Approach”, which was approved by the ethics committee of Bezmialem Vakif University (registration number: 15/10/2018-6032). Furthermore, informed consents of participants were obtained following the requirements of the research ethics boards. The blood samples were centrifuged at 3000 rpm for 15 min to separate the serum from the cellular component. Serum samples were then transferred into cryogenic tubes and stored at –80 °C until the infrared spectral data collection (Gajjar et al., 2013).

The samples were collected from 34 individuals at the age of 22–57, 12 of which were vaccinated and 22 of which were non-vaccinated. Healthy non-vaccinated individuals were considered as a control group. Demographic information is given in Table S1. Blood samples were taken 3–14 weeks after the second dose of CoronaVac in vaccinated group. 

### 2.2. ATR-FTIR spectroscopy

Frozen serum samples were thawed and vortexed at room temperature before spectroscopic analysis. The spectra were collected by a ALPHA II FTIR spectrometer (Bruker, USA) equipped with a Platinum ATR unit, which contains a diamond crystal. 1 µL of serum samples were placed on the crystal plate and dried with a very mild nitrogen gas flux for 2 min to remove excess unbound water. The spectra were collected in the 4000–650 cm^–1^ region at 4 cm^–1^ resolution, with 32 scan numbers. To eliminate the contribution of molecules in the air, first, prior to sample spectra acquisition, background spectrum of air was recorded under identical conditions as the samples and then subtracted automatically from all of the spectra. Recording of the spectra was performed using Opus 8.1 software (Bruker, USA) of the spectrometer. For each sample, three randomly taken replicates were scanned, which revealed almost identical spectra, and the spectral average of these replicates was used for further analysis. These average spectra were first smoothed with nine-point Savitsky–Golay smoothing function, interactive baseline corrected, and min-max normalized with respect to the amide A band for visual demonstration using Opus 5.5 software (Bruker, USA). The same software was also used to determine band positions (frequency) and band area values. The band positions were measured according to the center of mass of the width at 75% of band intensity (Yonar et al., 2018). The areas under the absorbance bands were calculated from the interactive baseline-corrected spectra.

### 2.3. Chemometrics

#### 2.3.1. Principal component analysis 

Principal component analysis (PCA) is an unsupervised method, which assists in the transformation of several comparable variables into a smaller number of different variables known as principal components (PCs). As a result, a smaller PC model can be used to quickly detect and discern abnormalities/deviations in the natural system (Jolliffe, 2002).

PCA was performed using The Unscrambler X 10.3 (CAMO Software AS, Norway) multivariate analysis (MVA) software. First, raw spectra of serum were preprocessed by using baseline offset transformation followed by peak normalization using the peak at 2959 cm^–1^ in the whole infrared region (4000–650 cm^–1^). The PCA model was formulated on the preprocessed data matrix after mean-centering and normalization of the spectra over 3050–2800 cm^–1^ spectral region. Nonlinear iterative partial least squares (NIPALS) algorithm and full cross validation was used by the PCA algorithm. The results are presented as scores and loadings plots.

#### 2.3.2. Linear discriminant analysis 

Linear discriminant analysis (LDA) is a supervised method. The PCA data were used as LDA model inputs using The Unscrambler X 10.3 (CAMO Software AS, Norway) multivariate analysis (MVA) software. The category variable column was included in a data matrix, and, subsequently, all spectra of different sample categories were used to build a training set. Quadratic classifiers method using projections of 7 PCA components were applied for prediction. Prior probabilities were calculated from the training set. The results are presented as a discrimination plot as well as classification and confusion matrices.

### 2.4. Statistics

Spectral results for all groups were expressed as mean ± standard error of the mean (SEM). The first normality test was applied to decide whether the parametric or nonparametric statistical test should be used. Since the data showed normal distribution, the vaccinated versus the non-vaccinated group were analysed using Dunnett’s multiple comparison test as a part of the one-way analysis of variance (ANOVA) in GraphPad Prism 6 (GraphPad Software Inc., San Diego, CA, USA). The degree of significance for the comparison of the vaccinated group with respect to the control group was denoted as * p < 0.05. 

## 3. Results and discussion

It is well known that infrared spectroscopy coupled with multivariate analysis techniques is widely used in disease diagnosis e.g., differentiation of healthy and sick individuals and in determination of the effectiveness of drug therapy (Gurbanov et al., 2016; Yonar et al., 2018; Heidari, 2020; Zhang et al., 2021; Barauna et al., 2021). Here, in this proof of concept study, we have used this capacity of infrared spectroscopy to determine CoronaVac-induced spectral variations in healthy human serum to better understand whether this vaccine causes biological changes in humans’ serum biomolecules or not. 

**Figure 1:**
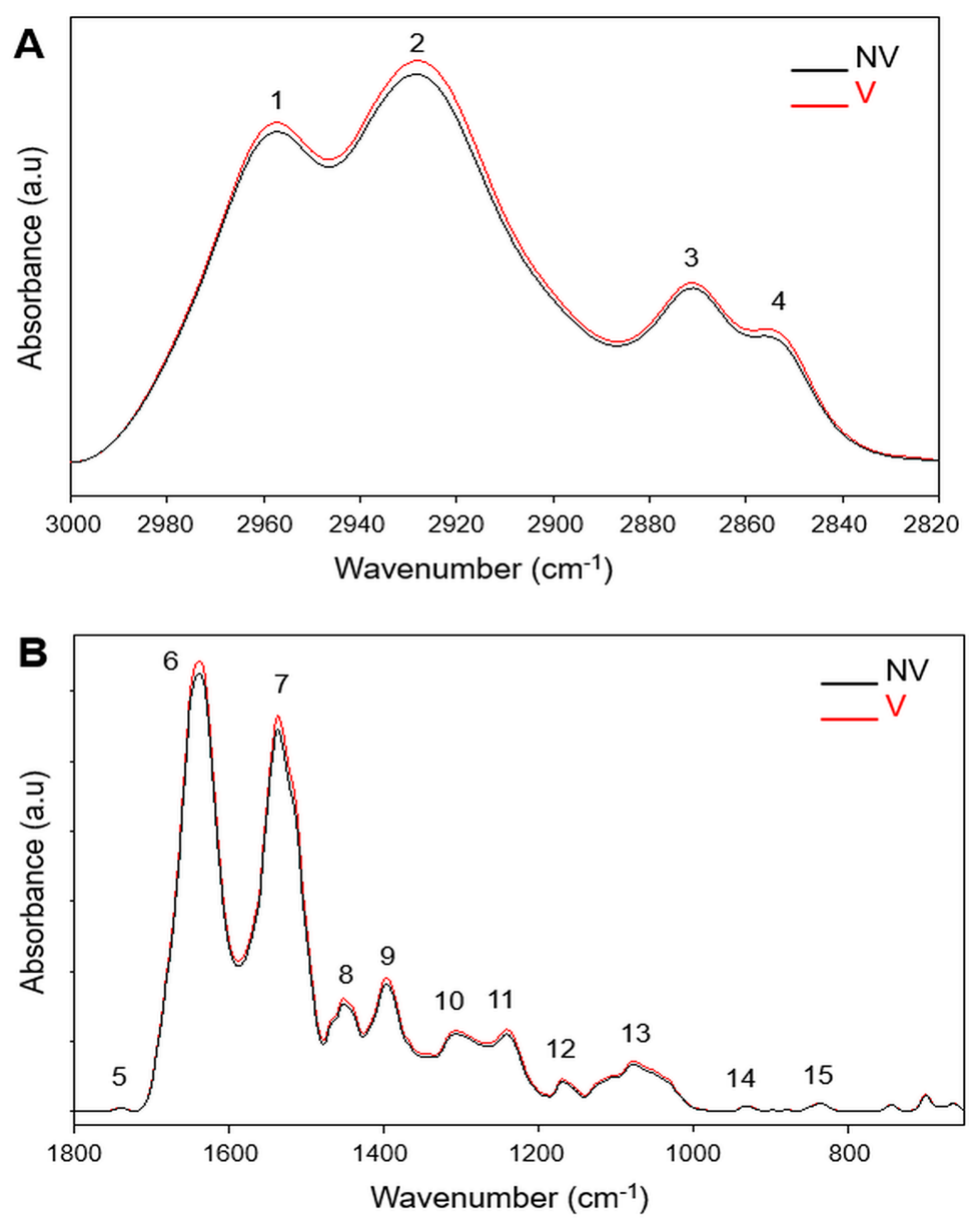
Average baseline-corrected infrared spectra of human serum from non-vaccinated (NV) and vaccinated (V) individuals in (A) the 3000–2800 cm–1 and (B) 1800–650 cm–1 regions. The spectra were normalized with respect to the amide A band at 3283 cm–1.

Figures 1A and 1B show the average infrared spectra of vaccinated and non-vaccinated groups in the 3000–2820 cm^–1^ and 1800–650 cm^–1^regions, respectively. The spectra were normalized with respect to the amide A band at 3283 cm^–1 ^position (not shown in Figure 1 and Table). Only the main peaks whose area values were calculated were labelled in Figure 1. The assignments of these bands are given in Table (Cakmak et al., 2011; Wood, 2016).

**Table:** The band assignment of an FTIR spectrum for human serum sample (Cakmak et al., 2011; Wood, 2016).

Band Number	Wavenumber (cm–1)	Band Assignment
1	2958	CH3 antisymmetric stretching: protein, lipid
2	2930	CH2 antisymmetric stretching: mainly lipid
3	2872	CH3 symmetric stretching: mainly protein
4	2853	CH2 symmetric stretching: mainly lipid
5	1738	Ester C=O stretching: trygliceride, cholesterol esters
6	1637	Amide I band mainly due to C=O stretching vibration of amide groups: protein
7	1537	Amide II band due to N-H bending strongly coupled to C-N stretching vibration of amide groups: protein
8	1453	CH2 bending mode of lipids
9	1396	COO- symmetric stretching: fatty acids and aminoacids
10	1307	Amide III: C–N stretching and N–H bending
11	1241	PO2– antisymmetric stretching: nucleic acid (B-form DNA) phospholipids
12	1170	C––OH asymmetric stretching: serine, treonine and tyrosine aminoacids of proteins
13	1079	C-O-C vibration of nucleic acids and phospholipids
14	931	Z- form DNA
15	836	A-form and B-form helix conformation of DNA

**Figure 2:**
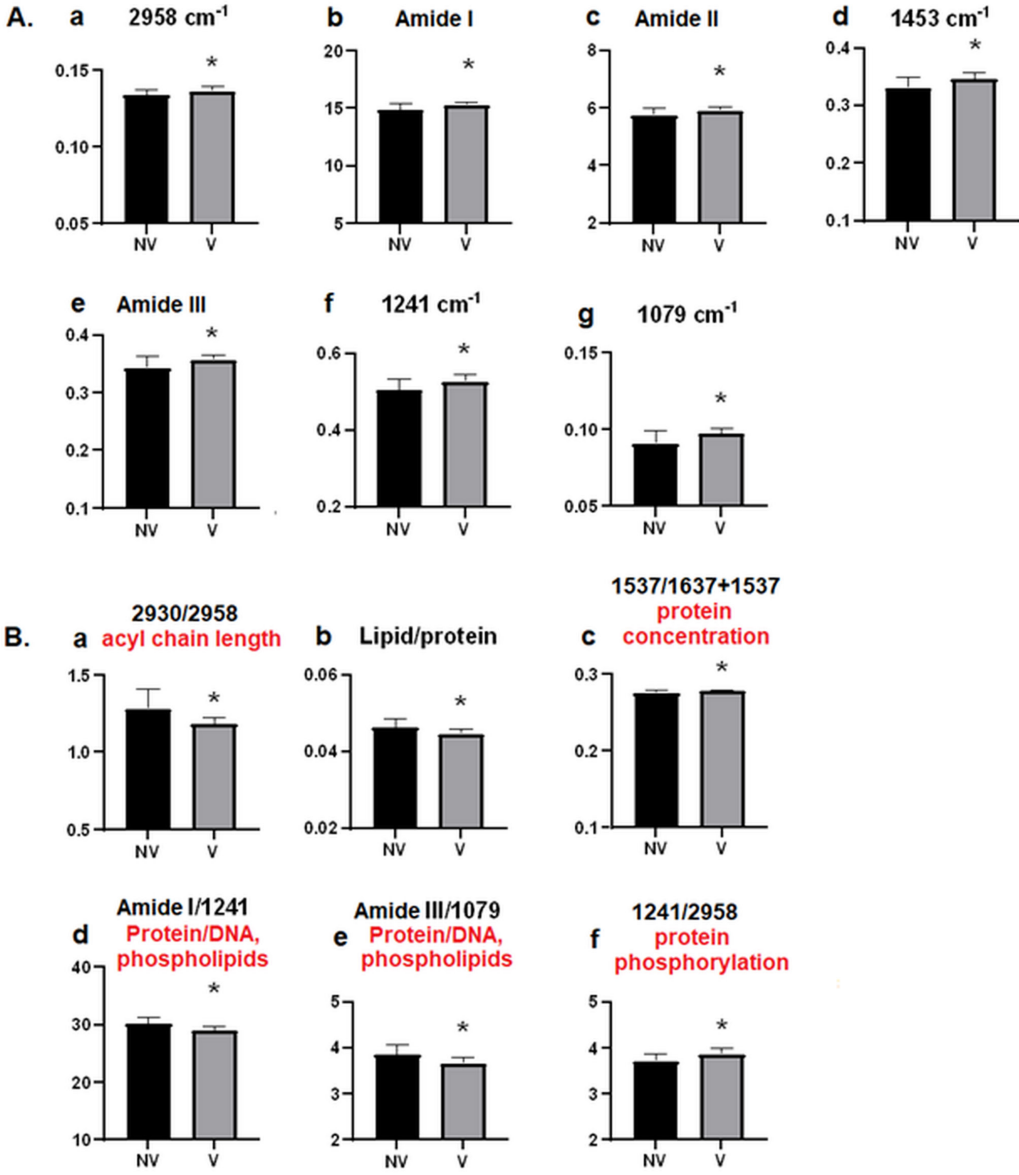
A. Changes in the band area values of (a) CH3 antisym. stretch. (2958 cm−1), (b) Amide I (1637 cm−1), (c) Amide II (1537 cm−1), (d) CH2 bending (1453 cm−1), (e) Amide III (1307 cm−1) (f) PO2 – antisym. stretch. (1241 cm−1), (g) PO2 – sym. stretch. (1079 cm−1) bands, and band area ratios of B. (a) Acyl chain length A2930 /A2958, (b) Lipid/protein A2958+2930+2853+1738+1453/A1637+1537+1307, (c) Protein concentration A1537/A1637+1537, (d) Protein/ nucleic acids, phospholipid A1637/A1241, (e) Protein/ nucleic acids, phospholipids, A1307/A1079, (f) Protein phosphorylation A1241/A2958 from the serum spectra of the non-vaccinated (NV) and vaccinated (V) individuals. The degree of significance was denoted as *p < 0.05.

According to Beer’s Lambart law, the intensity and/or the area of the infrared absorption bands rising from a particular functional group of the relevant molecule is directly proportional to the concentration of that molecule. Therefore, we used areas under the bands to obtain relative concentration information about biomolecules. These values are listed in Supplementary section as Table S2 and are given as bar diagrams in Figure 2.

In the C–H stretching region, the CH_2_ symmetric and antisymmetric stretching bands (band 2 and 4) arise from the lipid acyl chains and monitor saturated lipids. While the weak CH_3_ symmetric stretching band (band 3) is due to proteins, and the CH_3 _antisymmetric band (band 1) has an equal contribution from lipids and proteins (Table). 

As seen from Figure 1A, there is a slight variation in the absorbance of each band in the C-H stretching region between the compared groups. However, after the area calculation studies, a significant change is observed only in the CH_3 _antisymmetric stretching band (Figure 2A.a). The increase (p* < *0*.*05) in the area of this band suggests an increase in the lipid and/or protein content of the vaccinated group. This result was made clear by the area calculations of lipid and protein bands located at fingerprint region.

The fingerprint region between 1800–650 cm^–1 ^contains a series of infrared bands that belong to lipids, proteins, carbohydrates, and nucleic acids (Figure 1B, Table) (Balan et al., 2019). Analyses of this region showed that the areas of lipid band (band 8) located at 1453 cm^–1^ and protein bands namely amide I (band 6) at 1637 cm^–1^, amide II band (band 7) at 1537 cm^–1^ and Amide III band (band 10) at 1307 cm^–1^ increased significantly (Figure 2A). The increase in the protein amount was supported by the significant increase in the area ratios of amide II to amide I + amide II bands, which gives information about protein concentration more precisely due to the removal of experimental errors (Figure 2B.c). Since amide I band arises mainly from C=O stretching, amide II and amide III bands arise from N–H bending and C–N stretching of proteins; the change in the band areas of amide I, II and III may also indicate an alteration in the protein structure in the serum of the vaccinated (V) group (Cakmak et al., 2016). It is known that the concentration of immunoglobulin G (IgG), A (IgA), and M (IgM) elevate in viral infection (Santos et al., 2020). The study of Zhang et al. (2020) has demonstrated that the level of IgG antibodies in the patient serum increases after the onset of COVID-19. Therefore, increased protein amounts in vaccinated (V) groups may be seen because of immune system’s response by producing antibodies to  SARS-CoV-2 proteins. 

We have also observed a significant increase in PO_2_^–^ antisymmetric stretching at 1241 cm^–1 ^(Band 11) as shown in Figure 2A.f. This band is attributed to nucleic acids and phospholipids in serum. CoronaVac is an inactivated vaccine, which does not contain a live virus. Therefore, virus-induced change in the amount of nucleic acid is not expected. In addition, it is known that there are small amounts of nucleic acids such as cell-free circulating DNA (cirDNA) (Eccles 2005) or SARS-CoV-2 RNA (Wölfel et al., 2020) in human serum (Zang et al., 2021). Therefore, increased band area at 1241 cm^−1^ can be due to phospholipids. This finding is consistent with the literature. It is known that Coronavirus stimulates the B cell proliferation, which turns to plasma cell to increase Ig M level. The seroconversion of IgM and IgG take place around 12 days post onset of symptoms and neutralizing titers are seen on days 14–20 (Chvatal-Medina et al., 2021). At the end of this period, active neutralizing process occurs and plasma cells demolishe leading to release of cell contents including lipid, protein, and nucleic acid. In addition, it was recently reported that two main phospholipids, sphingolipids, and lysolecithin, in human serum were increased in COVID-19 patients (Wu et al., 2020; Zang et al., 2021). Furthermore, the significant incease that was observed in 1079 cm^–1^ band indicates oxydative damage in DNA (Mihoubi et al., 2017). 

We also found significant variations in the band area ratios of some specific bands. For example, the lipid acyl chain length can be calculated by taking the ratio of 2930 cm^–1^/2958 cm^–1^ bands (Figure 2B.a). (Gok et al., 2021). A decrease was observed in this ratio indicating an increase in shorter chain lipids in the vaccinated groups. The other ratios that are seen in Figure 2B imply the changes in biomolecular asymmetry. The ratio of the sum of the areas under the total lipid bands (2958 + 2930 + 2853 + 1738 + 1453) to the sum of the areas under the total protein (Amid I + II + III) bands was used to evaluate the lipid to protein ratio for serum samples. As seen from Figure 2B.b, the lipid/protein ratio decreased significantly in the vaccinated group (p < 0.05) compared to the non-vaccinated group. Since we did not observe significant variations in the main lipid bands (CH_2_ antisymmetric and symmetric) except the bending vibrations of lipids (band 8 at 1458 cm^–1^), this decrease could be attributed higher protein content. This result also indicates a variation in lipid to protein asymmetry (Cakmak et al., 2016). As seen Figure 2B.d, amide I to 1241 cm^–1^ ratio decreased significantly (p < 0.05) suggesting an increase in DNA concentration in the vaccinated group as also supported by an increase in the area of 1079 cm^–1^ DNA band (Figure 1 A.g). Supporting to the observed decrease in amide I / 1241 cm^–1^, a significant decrease was also observed in the band area ratio of protein amide III to 1079 cm^–1^ band (Figure.2B.e) (Ricciardi et al., 2017). The significant increase in the band area ratio of 1241 cm^–1^/2958 cm^–1^ (Figure.2B.f) indicating an increase in protein phosphorylation (Ricciardi et al., 2017), which is important in regulation of viral infection and many physiological processes, including signal transduction, regulation of transcription, survival and apoptosis (Nemes at al., 2017) . This result is consistent with the study of Bouhaddou et al., 2020, which revealed that Covid-19 causes increased phosphorylation on host and viral proteins. 

**Figure 3:**
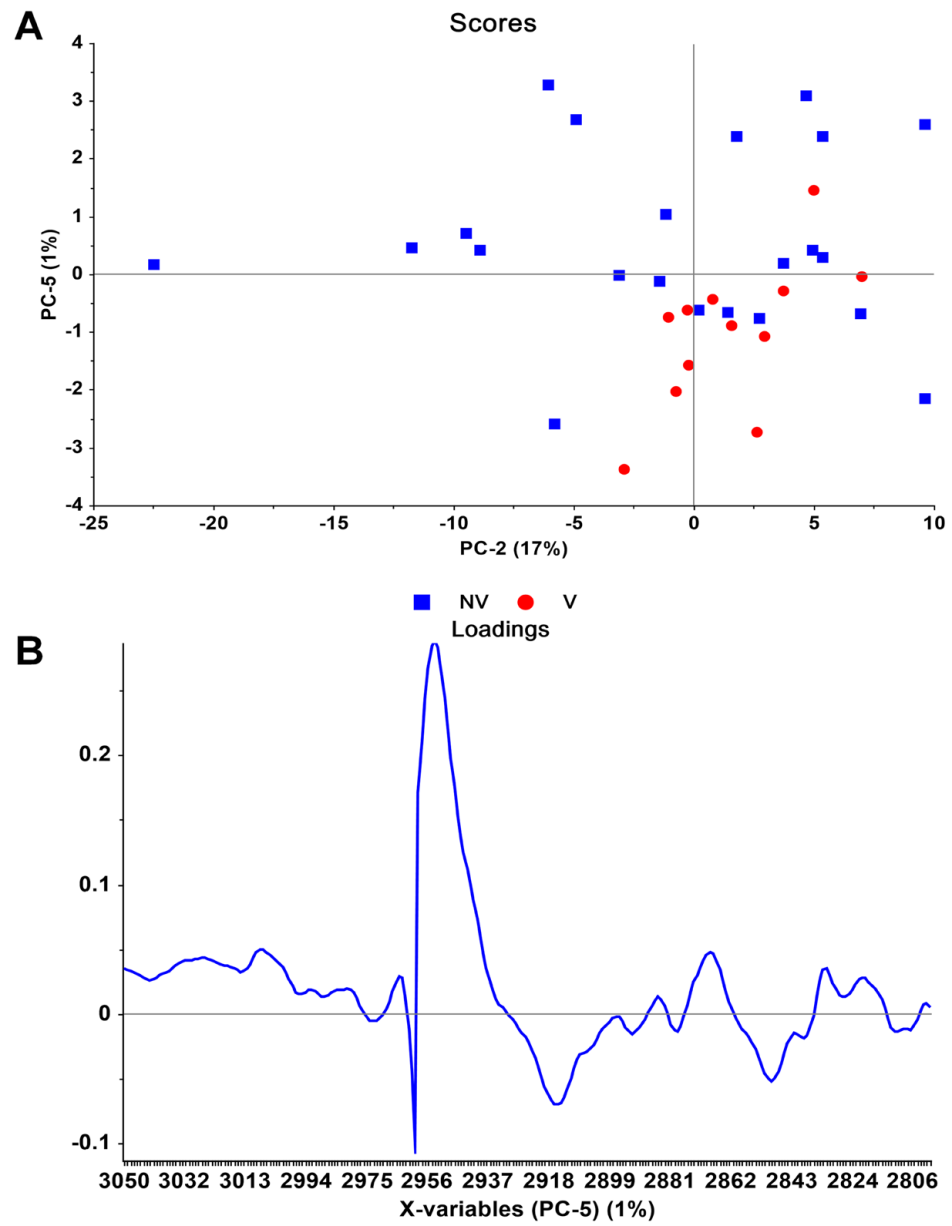
The differentiation between the serums obtained from non-vaccinated (NV) and vaccinated (V) individuals by unsupervised PCA. (A) Scores and (B) loadings plot were obtained in 3050–2800 cm−1 spectral region for NV and V groups.

**Figure 4:**
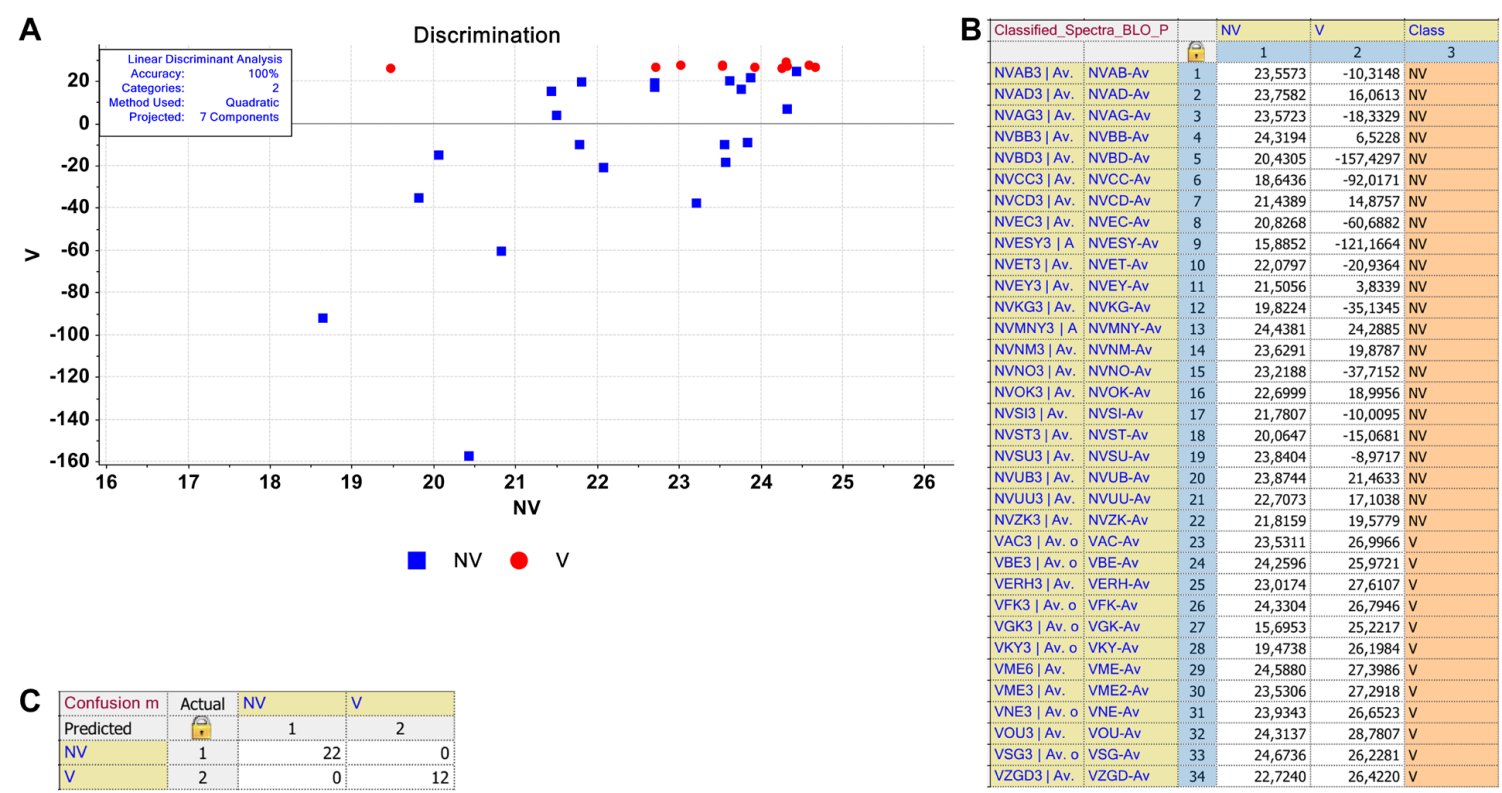
The discrimination of the serums obtained from non-vaccinated (NV) and vaccinated (V) individuals by supervised LDA. (A) The discrimination plot was obtained in 3050–2800 cm−1 spectral region for NV and V groups. (B) Classification and (C) confusion matrices of the model classes.

**Figure 5:**
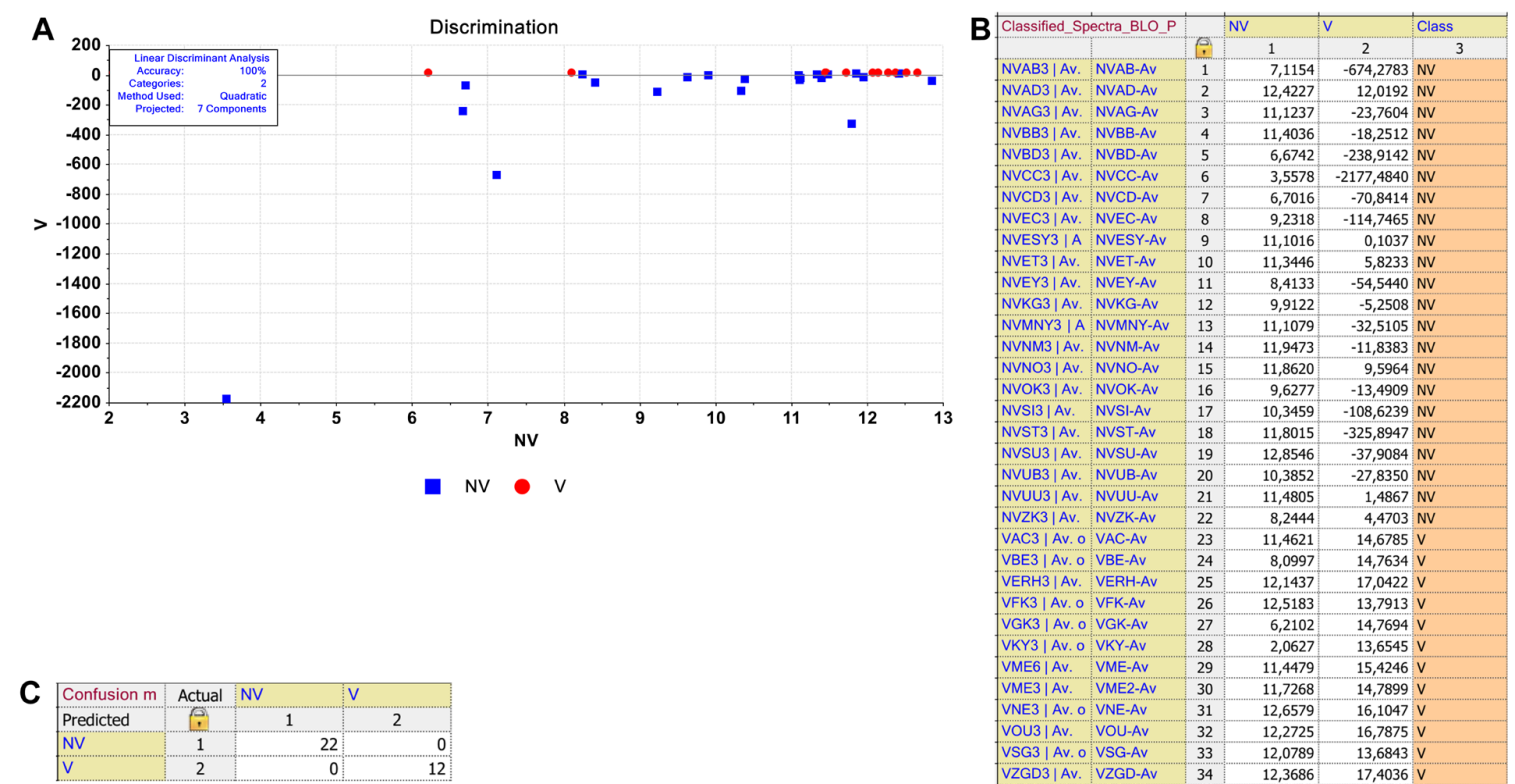
The discrimination of the serums obtained from non-vaccinated (NV) and vaccinated (V) individuals by supervised LDA. (A) The discrimination plot was obtained in 1300–800 cm−1 spectral region for NV and V groups. (B) Classification and (C) confusion matrices of the model classes.

Generally, due to complexity of the data, multivariate analysis methods are applied to spectral data to extract useful information from the data towards separation and discrimination of the groups. In this study, unsupervised PCA and supervised LDA chemometric methods were applied to the serum spectra of non-vaccinated (NV) and vaccinated (V) samples to see whether the two groups can be separated from each other or not. The developed PCA and LDA models are shown in Figures 3–5.

Figure 3A shows the PCA scores plot in the PC2-PC5 plane for the 3050–2800 cm^−1^ spectral region. Except for a few outliers, it can be observed that vaccinated group is separated from the non-vaccinated group, and the separation is mainly along the PC5 axis.  From the PCA scores plot, it is seen that 6 non-vaccinated individuals were located at the vaccinated group (PC5 < 0). This may be due to the existence of asymptomatic individuals. On the other hand, 1 vaccinated individual was found at the non-vaccinated group (PC5 > 0). The explained variances for PC2 and PC5 are 17% and 1%, respectively. This is an expected result: Overall spectrum is a superposition of a large number of peaks; serum samples are obtained from healthy individuals having different nature, and, therefore, high variations are expectable; on the other hand, the effect of vaccination creates only minor changes in a number of peaks, therefore, they will not have much effect on the scores along high variance principal components. In this case, all the principal components were checked, and separation of the two groups were observed only along the PC5 dimension. It is also known that loadings curve (eigenvectors) corresponding to different PCs are orthogonal to each other. Therefore, one can deduce the changes with respect to the mean spectrum, due to a PC, by multiplying the respective loadings curve with the corresponding score value. In this case, the scores along PC5 for vaccinated group have negative values, while scores for non-vaccinated group have positive values. It can, therefore, be concluded that, for the vaccinated group, there is a decrease at 2953 cm^–1^ and 2868 cm^–1^, and an increase at 2915 cm^–1^ and 2849 cm^–1^, relative to the normalized peak at 2959 cm^–1 ^(Figure 3B). In other words, these variables were found higher in the vaccinated group, compared with their average values. On the other hand, the negative correlation (opposite signs) between the positive discriminators, and the scores in the vaccinated group means that these variables are less than their corresponding average values in the vaccinated group. Both positive and/or negative correlations between scores and loadings plot indicate the alterations in the variable-represented biomolecules because of vaccination. 

LDA is a supervised classifier in which n-dimensional trait specimens are transformed into an m-dimensional space in a linear fashion (Gurbanov et al., 2019). While PCA uses only the sample spectra to determine the transformation, LDA, in addition, uses class information in the training samples. Therefore, it results in better classification. As shown in the discrimination plot, the supervised LDA model was developed with 100% accuracy for 3050–2800 cm^−1^ and 1300–800 cm^−1^spectral regions representing lipid and nucleic acid biomolecules, respectively (Figures 4A, 5A). However, other spectral regions also exhibited comparable model accuracies (data not shown). All the 22 non-vaccinated (NV) and 12 vaccinated (V) samples were correctly discriminated in their corresponding classes in the classification matrix, which shows each sample distance with respect to both classes (Figures 4B, 5B). According to the confusion matrix, the V class members were not confused with NV class members (Figures 4C, 5C).

The data obtained from infrared spectroscopy coupled with chemometric analyses showed biomolecular variations in the spectral signature of vaccinated groups serum. The results revealed that there are variations in the area of several bands such as 2915 cm^–1^ and 2849 cm^–1 ^absorptions in the direction of increase, and 2953 cm^–1^ and 2868 cm^–1 ^in the direction of decrease. However, detailed characterization studies showed that major spectral changes were obtained at 2958 cm^−1^, 1637 cm^−1^, 1537 cm^−1^, 1453 cm^−1^, 1307 cm^−1^, 1241 cm^−1^, and 1079 cm^−1 ^bands in the vaccinated group. The significant increase in the areas of these bands indicates higher lipid, protein and nucleic acid contents in the vaccinated groups. These findings enabled the successful PCA and LDA based discrimination of non-vaccinated and vaccinated groups from each other and suggest the power of ATR-FTIR spectroscopy as a sensitive test tool for diagnosis. 

Since blood samples of vaccinated individuals were taken in a period of 3–14 weeks after the vaccination, we belived that these molecular changes observed in the vaccinated group are induced by CoronaVac vaccine. It is important to note that the age interval of the individuals in this study is between 22–57. So, we present here the effect of CoronaVac vaccine on the humans who are young and middle age. 

The limitation of this proof of concept study is, first of all, the study’s quite low sample size. Also, as a public health measure, population groups above the age of 65 and below the age of 20 were subjected to strong lockdown regulations in Turkey. Their access to healthcare services were strictly limited. Therefore, obtaining blood samples especially from the age group above 65 was almost impossible. The recent total lockdown of 17 days contributed to the difficulty of providing samples. These limitations of access to study material can be expected to be lifted soon, as the restrictions due to the pandemic will not be lasting, and more samples from all age groups will be easily available.  

## 4. Conclusion 

Early diagnosis and treatment play a vital role in disease outbreaks. Vaccines are one of the most effective tools to prevent disease risk. However, vaccine design cannot always be achieved quickly due to technological difficulties as experienced in the COVID-19 pandemics. The advantages of FTIR spectroscopy as giving early diagnostic information (Toyran et al., 2006; Gok et al., 2016) in addition to its being low-cost, rapid, operator-independent, accurate, and being easy to use and requiring very little amount of sample e.g., one drop of serum, make this technique a good promising candidate in coronavirus diagnosis (Gok et al., 2016). Two infrared papers appeared recently in the literature about the fast and early diagnosis of COVID-19 (Barauna, 2021; Zhang et al., 2021). In one of those studies, it was reported that COVID-19 was successfully discriminated from influenza (Zhang et al., 2021). In the current study, we applied ATR-FTIR spectroscopy coupled with chemometrics to determine CoronaVac–induced changes in healthy human serum and observed that the vaccine causes significant changes in some functional groups belonging to lipids and proteins. Based on the spectral differences using chemometric techniques, the non-vaccinated and vaccinated groups were successfully separated from each other. This proof of concept study will stimulate future studies on CoronaVac and other vaccines with high human serum sample sizes and will be able to make a comparison between different vaccines. In addition, since vaccinated and non-vaccinated groups can be separated accurately, more rapidly and with lower cost than the gold standart methods, this low-cost approach can have a practical value and can be applicable in some places such as airports to get quick test results compared to gold standart methods. 
